# Personalized nutrition in haemodialysis: a scoping review of studies published between 2015 and 2025

**DOI:** 10.1093/ckj/sfag117

**Published:** 2026-04-16

**Authors:** José Francisco Rojas-Pérez, Sheila González-Salvatierra, Alejandro Oncina-Cánovas, Marina Padial, Verónica López-Jiménez, Gabriel Olveira

**Affiliations:** Diaverum Málaga, Málaga, Spain; IBIMA Plataforma BIONAND, Instituto de Investigación Biomédica de Málaga, Málaga, Spain; Department of Endocrinology and Nutrition, Hospital Regional Universitario de Málaga, Málaga, Spain; Instituto de Salud Carlos III, CIBER of Frailty and Healthy Aging (CIBERFES), Madrid, Spain; Instituto de Investigación Sanitaria y Biomédica de Alicante (ISABIAL), Alicante, Spain; Unidad de Epidemiología de la Nutrición, Departamento de Salud Pública, Historia de la Ciencia y Ginecología, Universidad Miguel Hernández (UMH), Alicante, Spain; Instituto de Salud Carlos III, CIBER of Epidemiology and Public Health (CIBERESP), Madrid, Spain; IBIMA Plataforma BIONAND, Instituto de Investigación Biomédica de Málaga, Málaga, Spain; Department of Endocrinology and Nutrition, Hospital Regional Universitario de Málaga, Málaga, Spain; Department of Medicine and Dermatology, University of Málaga, Málaga, Spain; IBIMA Plataforma BIONAND, Instituto de Investigación Biomédica de Málaga, Málaga, Spain; Department of Medicine and Dermatology, University of Málaga, Málaga, Spain; Department of Nephrology, Hospital Regional Universitario de Málaga, Málaga, Spain; Instituto de Salud Carlos III, National Network for Kidney Research RICORS2040 RD21/0005/0012, Madrid, Spain; IBIMA Plataforma BIONAND, Instituto de Investigación Biomédica de Málaga, Málaga, Spain; Department of Endocrinology and Nutrition, Hospital Regional Universitario de Málaga, Málaga, Spain; Department of Medicine and Dermatology, University of Málaga, Málaga, Spain; Instituto de Salud Carlos III, CIBER of Diabetes and Associated Metabolic Diseases (CIBERDEM), Madrid, Spain

**Keywords:** dietary adherence, haemodialysis, morphofunctional nutritional assessment, patient-centred care, personalized nutrition, protein-energy wasting

## Abstract

**Background:**

Protein-energy wasting, chronic inflammation, and functional decline are prevalent among patients undergoing haemodialysis (HD) and are associated with adverse outcomes and reduced quality of life. Although a substantial body of literature exists on nutritional management in HD, evidence has evolved considerably in recent years. Nutritional care in HD remains inconsistent and is limited by restrictive dietary paradigms and organizational barriers.

**Objective:**

To map evidence published between 2015 and 2025 on nutritional management in adult patients undergoing HD, focusing on personalized strategies, barriers to effective nutritional care, and patient-centred, function-oriented implementation.

**Methods:**

A scoping review was conducted following Joanna Briggs Institute methodology and reported according to PRISMA Extension for Scoping Reviews. PubMed/MEDLINE, Scopus, Web of Science, and Europe PMC were searched for English-language studies published between January 2015 and August 2025.

**Results:**

A total of 30 studies were included. The literature describes diverse personalized nutritional approaches, including oral and intradialytic supplementation, plant-forward dietary patterns, microbiota-oriented strategies, and targeted nutrient supplementation. Reported outcomes included nutritional biomarkers, inflammation, body composition, functional measures, and patient-reported experience. Key barriers to effective nutritional care were poor dietary adherence, psychosocial burden, limited health literacy, inconsistent professional guidance, and organizational constraints. Morphofunctional assessment tools provided added value beyond biochemical parameters, and the studies highlighted specific considerations for nutritional risk assessment in older adults undergoing HD.

**Conclusions:**

This scoping review highlights a shift towards more personalized and function-oriented nutritional care in HD, while underscoring persistent barriers and substantial evidence heterogeneity. The findings support future research and the development of more integrated, patient-centred, and sustainable nutritional care models.

## GRAPHICAL ABSTRACT

**GRAPHICAL ABSTRACT:**
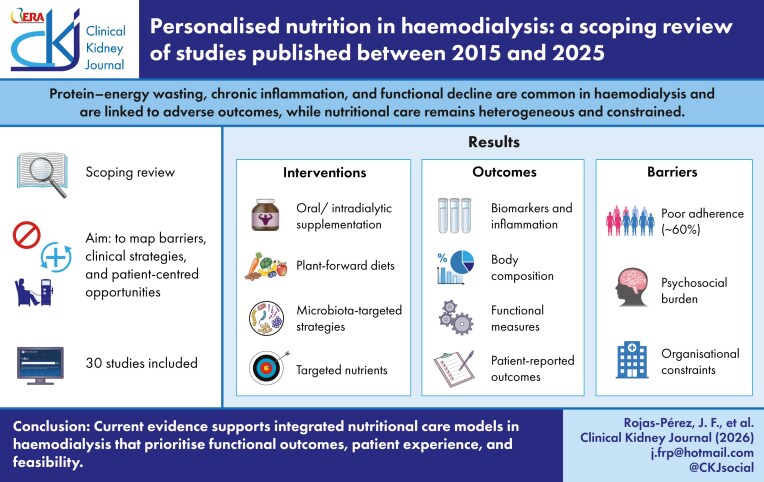

## INTRODUCTION

Chronic kidney disease (CKD) is a progressive and multifactorial condition that affects >10% of the global population and represents one of the leading causes of mortality worldwide [1–4]. Progression to end-stage renal disease (ESRD) requires renal replacement therapy (RRT), with haemodialysis (HD) remaining the most widely used modality, accounting for ∼69% of RRT worldwide [5, 6].

**Figure 1:**
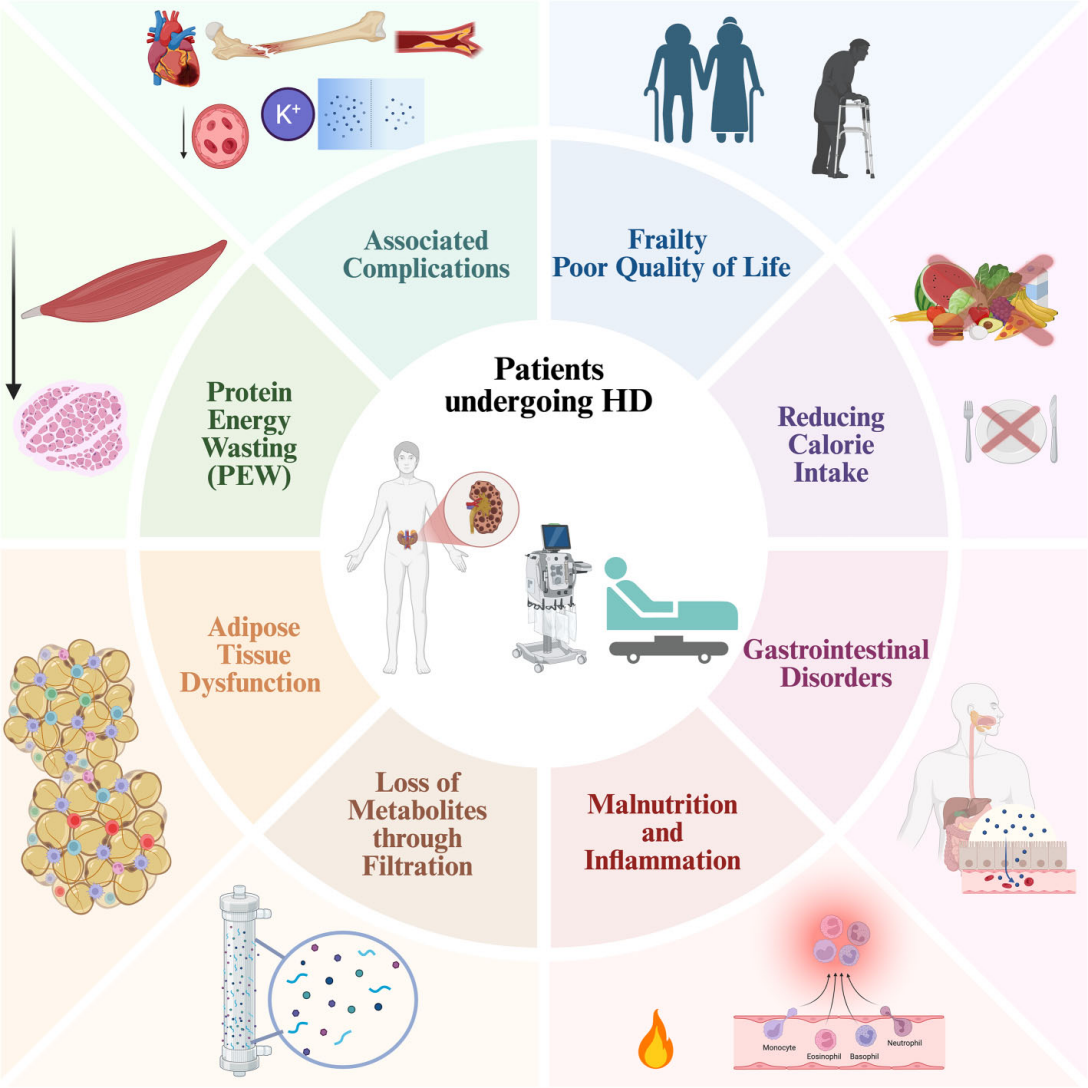
A schematic representation of the complex morbid-metabolic network affecting the nutritional status of patients undergoing HD. Alterations related to CKD, including PEW, adipose tissue dysfunction, metabolic derangements secondary to dialysis-related losses, gastrointestinal disorders, and reduced dietary intake, interact with systemic inflammation and malnutrition. These processes are described as contributing to functional impairment, frailty and poor quality of life. These interrelated pathways emphasize the multifactorial nature of nutritional deterioration in patients undergoing HD and highlight the importance of integrated, patient-centred nutritional and clinical management strategies. Created by the authors using BioRender.com.

Despite advances in dialysis technology and biocompatible materials, HD continues to be associated with high morbidity and mortality [7]. Patients undergoing HD experience a broad range of metabolic, cardiovascular, and nutritional complications that substantially influence prognosis, functional status, and quality of life [8–10] (Fig. 1). Among these, malnutrition, or protein-energy wasting (PEW) is one of the most prevalent and clinically relevant, affecting between 28% and 54% of patients undergoing HD [11, 12].

PEW is characterized by the loss of fat-free mass and body energy reserves resulting from inadequate dietary intake, increased protein catabolism, and persistent inflammation [11]. In the HD setting, PEW arises from the interaction of multiple mechanisms, including dialysis-related nutrient losses, chronic systemic inflammation, hormonal disturbances affecting appetite and energy metabolism, gastrointestinal symptoms, and psychosocial factors that reduce dietary intake and adherence [13–21]. These processes contribute to a self-perpetuating cycle of inflammation, catabolism, and muscle wasting, commonly referred to as the malnutrition–inflammation–atherosclerosis syndrome, which has been closely linked to sarcopenia, frailty, and increased mortality in HD populations [22–24]. Consequently, malnutrition in HD has been consistently associated with higher hospitalization rates, reduced treatment tolerance, and poorer survival [12, 20, 23].

Accurate identification of nutritional risk in HD remains challenging. Alterations in body composition, fluid shifts, and the frequent coexistence of sarcopenic obesity may obscure nutritional impairment, leading conventional indicators such as serum albumin or body mass index (BMI) to underestimate true risk [25–28]. In this context, morphofunctional assessment has emerged as a more comprehensive approach, integrating body composition, functional parameters, and inflammatory status to better reflect clinically relevant nutritional vulnerability [29, 30].

Alongside advances in assessment, personalized nutritional interventions, such as oral supplementation, intradialytic parenteral nutrition (IDPN), and individualized dietary education, have been associated with improvements in biochemical markers, body composition, functional outcomes, and quality of life [31–37]. However, despite this growing evidence base, the implementation of personalized and function-oriented nutritional strategies in routine HD care remains limited and inconsistent [12, 36]. This may partly reflect the absence of a shared conceptual framework defining personalized nutritional care in HD. Multiple terms are used interchangeably in the literature without clear consensus, and their specific application to HD contexts remains insufficiently articulated despite the population’s unique metabolic and functional challenges [38, 39]. Within this review, personalized nutrition in HD is defined as tailoring dietary strategies to an individual’s clinical phenotype, morphofunctional status, metabolic profile, and personal preferences, integrating nutritional intervention, functional assessment, and behavioural dimensions within a patient-centred framework [34, 40–43].

Most existing reviews focus on isolated dietary components, supplementation strategies, or biochemical outcomes, often without considering the psychosocial, organizational, and methodological factors that determine real-world effectiveness. Moreover, nutritional care in HD continues to be largely restrictive and laboratory-driven, with limited integration of functional assessment, patient experience, and heterogeneity of nutritional risk, particularly among older and frailer patients.

Given the conceptual and methodological diversity of the available evidence, this work was designed as a scoping review. To reflect current advances in this field, this review has focused on recently published studies, which reflect the evolution of clinical practice and emerging conceptual frameworks in the nutritional care of HD. The primary aim was to synthesize evidence published between 2015 and 2025 on nutritional management in adult patients undergoing HD, with a specific focus on personalized nutritional strategies, barriers to effective nutritional care, and opportunities for patient-centred, function-oriented implementation. By adopting this implementation-focused perspective, the review seeks to characterize prevailing practices, identify knowledge gaps, and inform future research and organizational strategies in HD nutrition.

## MATERIALS AND METHODS

This study was conducted as a scoping review to systematically map and synthesize the heterogeneous evidence on nutritional management in adult patients undergoing HD. A scoping review approach was considered appropriate given the substantial variability in study designs, populations, interventions, outcomes and follow-up durations, which precluded quantitative synthesis or meta-analysis.

The review was conducted in accordance with the Joanna Briggs Institute methodology for scoping reviews [44] and reported following the PRISMA Extension for Scoping Reviews (PRISMA-ScR) [45]. The PRISMA-ScR checklist is provided as Supplementary Table S1. The protocol was prospectively registered in the Open Science Framework (DOI: 10.17605/OSF.IO/QM3PK).

### Information sources and search strategy

A comprehensive literature search was performed on 10 August 2025 in four electronic databases: PubMed/MEDLINE, Scopus, Web of Science, and Europe PMC. These databases were selected to ensure broad coverage of biomedical, nephrology, and clinical nutrition literature.

The search strategy combined controlled vocabulary terms and free-text keywords related to HD and nutritional management, including terms such as ‘*hemodialysis’, ‘haemodialysis’, ‘renal dialysis’, ‘nutrition therapy’, ‘nutritional management’, ‘dietary intervention’*, and *‘malnutrition.’* The strategy was initially developed for PubMed/MEDLINE and subsequently adapted to the syntax of the remaining databases.

Searches were limited to human studies published in English between January 2015 and August 2025, focused on title, abstract, or keywords. This time frame was selected to capture contemporary evidence reflecting current HD practices, nutritional assessment tools, and intervention strategies. The full search strategies for each database are provided in Supplementary information 1 and Supplementary Table S2.

### Eligibility criteria

Eligibility criteria were defined using the Population–Concept–Context framework. The population comprised adult patients (≥18 years) undergoing maintenance HD. The concept encompassed nutritional care, including nutritional assessment, dietary counselling, oral and intradialytic supplementation, dietary adherence, patient experience, and personalized nutritional strategies, as well as reported barriers and facilitators to implementation. The context was outpatient HD care across diverse healthcare settings.

Personalized nutrition was defined broadly as any nutritional strategy adapted to at least one individual characteristic, such as nutritional status, comorbidities, biochemical profile, functional capacity, patient preferences, or behavioural factors.

Accordingly, the following research questions guided the review:

- What nutritional strategies have been explored for adult patients undergoing HD?
- What barriers and facilitators to effective nutritional care in HD have been reported?
- What nutritional assessment tools are used to identify nutritional risk and to guide personalized, patient-centred nutritional care in this population?

Eligible publications included original quantitative, qualitative, or mixed-methods studies, as well as systematic, narrative, and scoping reviews addressing nutrition-related aspects of HD care. Studies focusing exclusively on peritoneal dialysis, kidney transplantation, acute kidney injury, paediatric populations, or nonclinical contexts were excluded, as were editorials, commentaries, conference abstracts, and opinion papers without original data.

### Study selection

**Figure 2:**
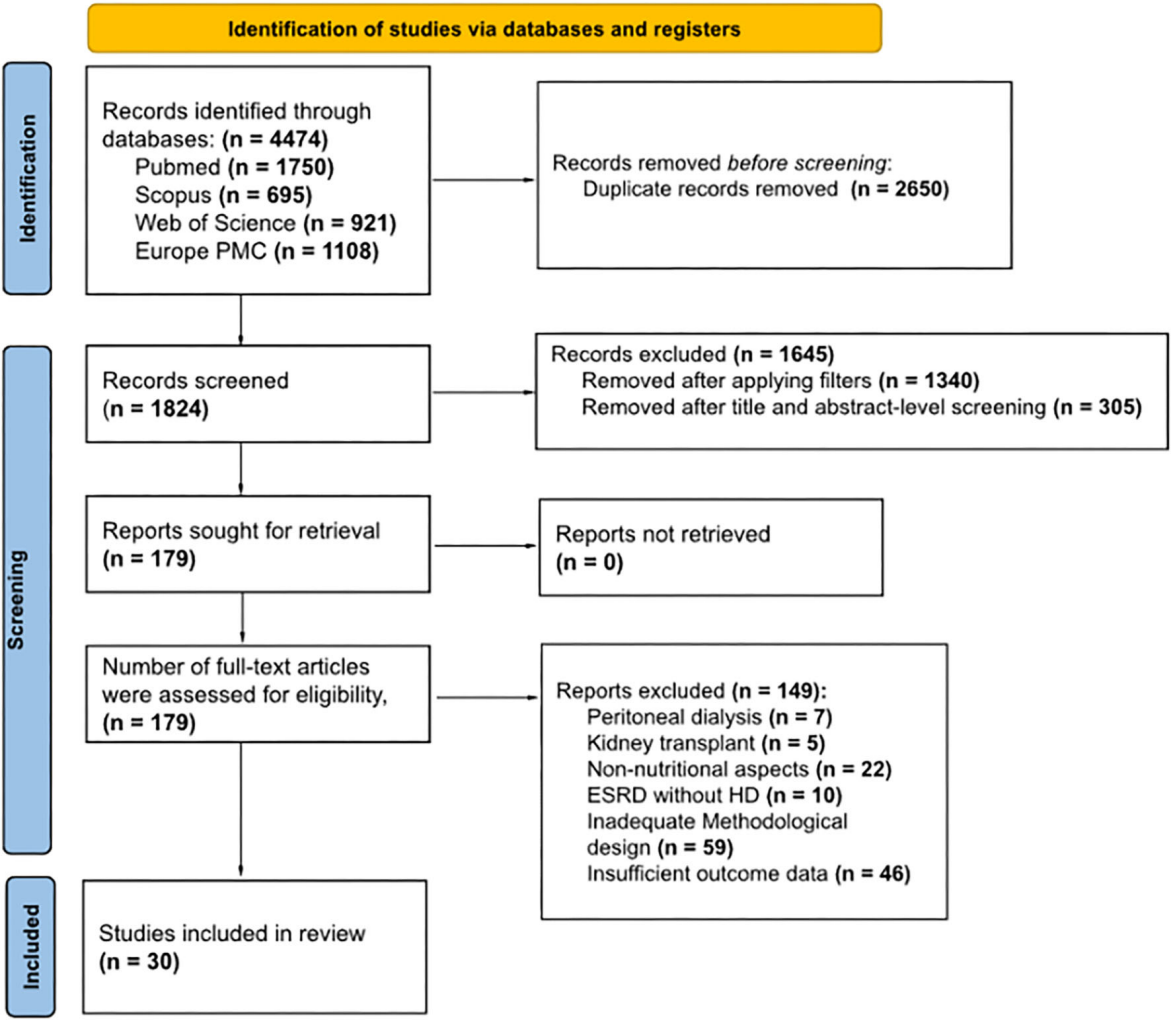
PRISMA flow chart describing the identification, screening, and selecting process of studies published between 2015 and 2025 included in the scoping review (*n* = 30).

Study selection followed PRISMA-ScR recommendations [45]. All records were imported into Zotero for reference management and duplicate removal and subsequently uploaded to Rayyan [46] for screening. Titles and abstracts were independently screened by two reviewers, followed by full-text assessment of potentially eligible articles. Discrepancies were resolved by discussion and consensus. The selection process and reasons for exclusion are summarized in the PRISMA flow diagram (Fig. 2).

### Data charting and synthesis

Data charting was performed using a predefined extraction framework aligned with the objectives of the review. Extracted data included study design, sample size, clinical setting, nutritional assessment tools, characteristics of nutritional interventions, outcomes assessed, and relevance to clinical practice.

Consistent with the exploratory nature of a scoping review, no formal risk-of-bias scoring was applied. Instead, methodological characteristics were considered narratively, guided by established reporting frameworks according to study type (STROBE [47] for observational studies, CONSORT [48] for clinical trials, and AMSTAR 2 [49] for review articles), without quantitative appraisal. Additional methodological details are provided in Supplementary information 2.

Data synthesis followed a descriptive and thematic approach. Studies were organized into three predefined analytical domains: (i) nutritional interventions and supplementation strategies; (ii) dietary adherence and patient experience; and (iii) nutritional assessment approaches, including considerations for older adults undergoing HD. The synthesis prioritized clinical relevance and implementation-related insights over effect size estimation. Detailed findings are reported in the Results section.

In addition, reported outcomes were descriptively categorized according to their level of clinical translation to enhance interpretative clarity. Outcomes were grouped into three categories: (i) hard clinical outcomes reflecting broader disease trajectory (e.g. hospitalization, dialysis tolerance, mortality, or prevalence of PEW); (ii) surrogate or intermediate markers including biochemical, inflammatory, and morphofunctional parameters [e.g. serum prealbumin, C-reactive protein, muscle ultrasound, and malnutrition–inflammation score (MIS)]; and (iii) patient-reported outcomes encompassing experiential and behavioural dimensions such as quality of life, appetite, vitality, and dietary adherence. This classification was applied descriptively and did not imply hierarchical weighting.

## RESULTS

A total of 4474 records were identified across PubMed, Scopus, Web of Science, and Europe PMC. After removal of duplicates, 1824 records were screened by title and abstract, leading to the exclusion of 1645 studies that did not meet the predefined inclusion criteria. Full-text assessment was performed for 179 articles, of which 149 were excluded mainly due to non-HD populations, lack of a nutritional intervention or focus, inadequate study design, or insufficient outcome reporting. Ultimately, 30 studies were included in this scoping review (Fig. 2).

The included studies were selected for their direct relevance to nutritional management in adult patients undergoing HD, with a particular focus on personalized nutritional strategies, barriers to effective care, and implementation-related considerations. This thematic focus allowed the synthesis of clinically meaningful evidence and the identification of key research and practice priorities in HD nutrition care.

### Characteristics of the included studies

Of the 30 studies included in this scoping review, 17 (56.7%) were primary research studies, encompassing observational designs (cross-sectional and cohort studies), interventional studies (randomized and non-randomized trials), and qualitative research. The remaining 13 studies (43.3%) were review articles, including systematic reviews, meta-analyses, narrative reviews, and integrative reviews. This distribution illustrates the methodological and conceptual heterogeneity of the literature addressing nutritional management in adult patients undergoing HD, with a substantial proportion of synthesized evidence focusing on dietary strategies, barriers to nutritional care, and implementation-related aspects. The methodological characteristics of the included studies are summarized in Supplementary Table S3.

Primary studies and reviews were analysed separately. Review articles informed overarching themes and conceptual approaches, whereas primary studies provided empirical data on clinical practices, patient experiences, and implementation-related outcomes (Tables 1-3).

**Table 1:** Summary and characteristics of studies on nutritional interventions.

Authors/year	Country (region)	Study design/sample	Intervention	Main outcomes	Clinical relevance
**Limwannata *et al*., 2021** [50]	Thailand (Asia)	RCT; *n* = 72	Renal-specific oral supplement vs. diet counselling(6 weeks)	↑ albumin, prealbumin, protein intake, dry weight; ↓ MIS	Oral renal-specific supplements outperform counselling alone for PEW management
**Elsayed *et al.*, 2025** [51]	Egypt (Africa)	Multicentre RCT; (*n* ≈ 100)	Predialytic protein-based oral supplements	↑ albumin, BMI, nPCR;↓ MIS; ↑ QoL	Predialytic supplementation improves nutrition and patient-reported outcomes
**Marsen *et al*., 2017** [52]	Germany (Europe)	Multicentre RCT; (*n* = 83) PEW	IDPN vs. standard care (16 weeks)	↑ prealbumin, nitrogen balance; good tolerance	IDPN beneficial for selected PEW patients with insufficient oral intake
**Arias-Guillén *et al.*, 2024** [36]	Spain (Europe)	Multicentre retrospective; (*n* = 56) PEW	IDPN (≥2 weeks)	↓ MIS, ↓ PEW prevalence; ↑ albumin, total protein	Confirms real-world safety and short-term effectiveness of IDPN
**Tsai *et al.*, 2023** [53]	Taiwan (Asia)	RCT crossover; (*n* = 34)	Low-inorganic P, plant-fibre therapeutic diet	↓ phosphorus, PTH and FGF-23; no hyperkalaemia	Structured plant-forward diets improve mineral metabolism safely
**González-Ortiz *et al.*, 2021** [54]	Mexico (North America)	Longitudinal cohort; (*n* = 108)	≥50% plant-based protein intake	Adequate protein intake; ↓ FGF-23; no ↑ K⁺	Supervised plant-based diets are nutritionally safe in patients undergoing HD
**Miyoshi *et al*., 2020** [55]	Japan(Asia)	Prospective intervention; (*n* = 10)	PHGG prebiotic (12 weeks)	Improved bowel function; ↑ SCFAs; favourable microbiota changes	Low-cost strategy for constipation and gut health in HD
**Chen *et al.*, 2023** [56]	China(Asia)(review; multinational included trials)	SR & meta-analysis; 18 RCTs, (*n* = 1352)	Probiotics/prebiotics/symbiotics	↓ CRP, IL-6 and uremic toxins; improved GI symptoms	Adjunct therapy for inflammation and dysbiosis; safe
**Liu *et al.*, 2022** [57]	China(Asia)	SR & meta-analysis; 12 RCTs, (*n* ≈ 796)	Omega-3 fatty acids	↓ triglycerides;↓ inflammatory markers	Supports omega-3 as adjunct cardiometabolic strategy
**Bogacka *et al.*, 2024** [58]	Poland (Europe)	Prospective intervention; (*n* = 64)	Antioxidant diet + vitamins C/E	↓ inflammation; ↑ antioxidant capacity; ↑ vitality	Feasible strategy to mitigate oxidative stress
**Bernales-Delmon *et al.*, 2025** [59]	Chile (South America)	Open-label trial; (*n* = 40)	Oral creatine(8 weeks)	↑ muscle mass; ↑ physical performance; safe	Promising anabolic adjunct for sarcopenia prevention

RCT: randomized controlled trial; HD: haemodialysis; MIS: malnutrition–inflammation score; PEW: protein–energy wasting; BMI: body mass index; nPCR: normalized protein catabolic rate; QoL: quality of life; IDPN: intradialytic parenteral nutrition; PTH: parathyroid hormone; FGF-23: fibroblast growth factor 23; K⁺: potassium; PHGG: partially hydrolysed guar gum; SCFAs: short-chain fatty acids; SR: systematic review; CRP: C-reactive protein; IL-6: interleukin-6.

**Table 2:** Summary and characteristics of studies on dietary adherence and patient experience.

Authors/year	Country (region)	Study design/sample	Focus/method	Key patient-centred findings	Clinical relevance
**Gebrie & Ford, 2019** [60]	Ethiopia (Africa)(review; multinational)	Systematic review; 12 studies(*n* ≈ 9000)	Depression and dietary nonadherence	Depressive symptoms strongly associated with diet/fluid nonadherence, poorer phosphorus and potassium control, and higher interdialytic weight gain (OR 1.8–3.2)	Psychological distress is a major driver of nonadherence; routine depression screening should accompany nutritional care
**Vr & Kang, 2022** [61]	India(Asia)(review; multinational)	Systematic review & meta-analysis; 23 studies (*n* = 11 209)	Global prevalence of nonadherence	Global nonadherence prevalence ≈60% for both diet and fluid restrictions	Nonadherence is widespread and requires multifactorial, patient-centred interventions beyond dietary prescription
**Hendriks *et al.*, 2021** [62]	Netherlands (Europe)	Narrative review	Protein intake, anabolic resistance, patient routines	Protein intake is lower on dialysis days due to fatigue, time constraints, and eating environment; amino acid losses (∼12 g/session) worsen catabolism and symptoms	Interventions must fit patient routines and address practical barriers, not only increase protein targets
**Rizk *et al.*, 2017** [63]	Lebanon (Asia)	RCT; (*n* = 110)	Stage-based education by renal dietitians	Improved phosphorus control (∼0.5 mg/dL), dietary adherence, self-efficacy, and perceived control compared with usual care	Dietitian-led, behaviour-oriented education improves biochemical control and patient empowerment
**Oquendo *et al.*, 2017** [64]	Spain (Europe)	Integrative review; 23 studies	Determinants of diet adherence	Barriers: emotional distress, rigidity of diet, cultural mismatch, symptoms, and low literacy; facilitators: clear communication, family support, and tailored education	Adherence improves when emotional, social, and cultural factors are addressed alongside clinical guidance
**Padial *et al.*, 2025** [65]	Spain (Europe)	Qualitative study; (*n* = 129) (pts + caregiv-ers)	Perceived needs, barriers, facilitators	Restrictive diet perception, fatigue, conflicting advice, and low literacy reduce adherence; understanding diet–lab links improves self-efficacy and compliance	Consistent messaging, caregiver involvement, and culturally adapted counselling are essential in routine care
**McLean *et al.*, 2021** [66]	New Zealand (Oceania)	Qualitative interviews; (*n* = 35)	Lived experiences with food management	Patients report confusion, frustration, fear, and cultural barriers; rely on family support and trial-and-error coping strategies	Nutritional care must address emotional burden and cultural context, not only technical recommendations
**Mesa-Gresa *et al.*, 2024**[67]	Spain; Belgium; Greece; Sweden (Europe)(multicentre)	Multicentre observational study; patients (*n* = 38), caregivers (*n* = 34), and healthcare professionals (*n* = 38).	Lifestyle needs, barriers, and facilitators in HD; exploratory structured questionnaires	Patients report difficulty understanding dietary recommendations (especially minerals), frustration with restricted food choices, and fatigue limiting meal preparation; personalized education and supportive communication were viewed as key facilitators	Highlights practical, cognitive, and emotional barriers to dietary adherence; emphasizes need for clear, tailored nutrition education and multidisciplinary support to sustain behaviour change
**Kanno *et al.*, 2021** [40]	Japan(Asia)	Narrative review	Nutritional strategies in HD	Continuous dietitian involvement, simplified communication, and stepwise education improve understanding and adherence	Sustainable nutrition care depends on ongoing, patient-centred education rather than restrictive rules
**Uribarri, 2018** [41]	United States (North America)	Narrative review	‘Aspirational diet’ concept	Overly restrictive renal diets impair quality of life; flexible, plant-forward, whole-food approaches may improve adherence without increasing risk	Encourages individualized, enjoyable diets guided by dietitians rather than rigid avoidance-based models
**Piccoli *et al.*, 2017** [39]	Italy(Europe)	Narrative review	Diet-HD dyad and paradoxes	Contradictory rules cause frustration and disengagement; personalized, flexible advice improves quality of life and intake	Shared decision-making and coherence among professionals enhance adherence and engagement
**Biruete *et al*., 2017** [68]	United States (North America)	Narrative review	Modified nutritional recommendations	Flexible restrictions and patient-centred strategies improve diet quality and satisfaction compared with traditional restrictive diets	Supports transitioning from rigid renal diets to individualized nutrition plans
**Zhang *et al.*, 2025** [69]	China(Asia)	Narrative review	PEW determinants and interventions	PEW driven by inflammation, anorexia, metabolic disturbances; combined nutrition, exercise, and anti-inflammatory strategies most effective	Highlights need for integrated, patient-tailored, multimodal interventions

RCT: randomized controlled trial; HD: haemodialysis; PEW: protein–energy wasting.

**Table 3:** Summary and characteristics of studies on nutritional assessment and considerations in older adult patients undergoing HD.

Authors/year	Country (region)	Study design/sample	Method/assessment tool	Key findings	Clinical relevance
**Hamdan *et al.*, 2025** [70]	Palestine (Asia)	Cross-sectional study; (*n* = 180)	MIS, dietary intake, inflammatory markers, clinical variables	MIS > 8 associated with ↑CRP (+36%), ↓ albumin (−18%), higher PEW prevalence (72%), poorer diet and longer HD vintage	MIS is a robust integrative marker of nutritional-inflammatory burden; identifies high-risk patients requiring targeted intervention
**Sabatino *et al.*, 2019** [71]	Italy (Europe)	Cross-sectional observational; (*n* = 96)	Ultrasound of rectus femoris and vastus intermedius; comparison with DXA and BIA	Strong correlation with DXA (*r* = 0.82) and BIA (*r* = 0.74); muscle thickness <13 mm linked to 2.3-fold, ↑ hospitalization/mortality	Muscle ultrasound is feasible, noninvasive and predictive of adverse outcomes, even when BMI is normal
**Matsuzawa *et al.*, 2021** [72]	Japan (Asia)	Diagnostic accuracy study; (*n* = 148)	Quadriceps ultrasound vs. handgrip strength, physical performance and BIA	Sensitivity 83%, specificity 78%; strong correlation with handgrip (*r* = 0.68); lower thickness in sarcopenic patients	Supports ultrasound as a reliable diagnostic tool for sarcopenia with functional relevance
**Ishida & Kato, 2023** [73]	Japan (Asia)	Narrative review	Review of SGA, MIS, GNRI, BIA, ultrasound	Traditional markers insufficient; increasing use of MF-BIA and ultrasound; PEW prevalence 20%–40%	Endorses a multimodal, standardized nutritional assessment combining morphofunctional tools
**Rodrigues *et al.*, 2017** [74]	Brazil (South America)	Narrative review	Critical review of anthropometry, labs, functional and body composition tools	BMI and albumin underestimate malnutrition; sarcopenia and frailty frequently missed	Recommends adapted criteria and integration of functional and morphofunctional measures in older adults
**Martins *et al.*, 2017** [75]	Brazil (South America)	Cross-sectional study; (*n* = 130)	Dietary recalls, food frequency, ultraprocessed food intake	Patients undergoing HD had ↓ protein intake (−25%), ↑ ultraprocessed foods (+40%), PEW 61% and frailty 48%	Highlights the interaction between poor diet quality and muscle deterioration, reinforcing need for tailored assessment

MIS: malnutrition–inflammation score; CRP: C-reactive protein; PEW: protein-energy wasting; HD: haemodialysis; DXA: dual-energy X-ray absorptiometry; BIA: bioelectrical impedance analysis; BMI: body mass index; SGA: subjective global assessment; GNRI: geriatric nutritional risk index; MF-BIA: multifrequency bioelectrical impedance analysis.

Interventional studies were generally small and short-term, most enrolling fewer than 100 participants with follow-up periods of 6–16 weeks. Observational studies were predominantly cross-sectional and focused on nutritional status, dietary intake, inflammatory markers, body composition, and functional outcomes. Qualitative studies explored patient and caregiver perspectives on nutritional care in HD.

The included studies were published between 2015 and 2025 and originated from diverse geographical regions, including Asia (*n* = 12), Europe (*n* = 9), North America (*n* = 3), South America (*n* = 3), Africa (*n* = 2), and Oceania (*n* = 1), reflecting a broad international distribution of evidence. Studies were mainly conducted in outpatient HD units, with considerable variability in healthcare organization, availability of renal dietitians, and the degree of integration of multidisciplinary nutritional care into routine clinical practice. Population characteristics and the geographic distribution of the included studies are summarized in Supplementary Tables S4 and S5.

### Nutritional interventions and supplementation strategies in patients undergoing HD

Eleven studies evaluated the effects of nutritional interventions in patients undergoing HD (Table 1), including individualized oral supplementation, IDPN, structured dietary approaches, gut microbiota-oriented strategies, and targeted nutrient supplementation [36, 50–59].

Individualized oral protein-energy supplementation was assessed in two randomized controlled trials involving patients with PEW [50, 51]. Both home-based and predialytic supplementation strategies were associated with improvements in nutritional biomarkers, dietary intake, MIS, and patient-reported outcomes, without relevant adverse effects. IDPN was examined in randomized and observational studies in patients with PEW or insufficient oral intake [36, 52], showing improvements in selected nutritional and inflammatory markers, although effects on anthropometric outcomes were inconsistent.

Structured dietary interventions, including low-inorganic phosphorus, high-fibre diets, and plant-based dietary patterns, were explored in two studies [53, 54]. These approaches were associated with improved phosphorus-related parameters, maintenance of protein intake adequacy, and favourable microbiota-related outcomes, without an increased risk of hyperkalaemia.

Gut microbiota modulation through prebiotics, probiotics, and symbiotics was examined in two studies [55, 56], reporting changes in bowel-related outcomes, inflammatory markers, and uraemic toxins, with a favourable safety profile. Omega-3 fatty acid supplementation and antioxidant-based dietary interventions were also evaluated [57, 58], with reported effects on lipid metabolism, inflammation, oxidative stress markers, and selected patient-reported outcomes.

Finally, one open-label trial assessed oral creatine monohydrate supplementation [59] describing improvements in functional capacity and body composition parameters without significant safety concerns.

Overall, the available evidence suggested that personalized nutritional strategies in HD were explored across multiple clinical domains, including nutritional status, inflammation, body composition, and functional outcomes. However, substantial heterogeneity in study design, intervention protocols, and outcome reporting limited comparative interpretation and precluded firm evidence-based recommendations.

### Dietary adherence and the experience of patients undergoing HD in nutritional management

Thirteen studies addressed dietary adherence and patient experience in nutritional management during HD (Table 2) [39–41, 60–69]. Overall, nonadherence to dietary and fluid recommendations was consistently reported, with a global prevalence of ∼60%, although substantial heterogeneity was observed across settings and assessment methods [61].

Several studies highlighted the role of personalized nutritional education and psychosocial support in improving adherence [60–64]. Depression, low health literacy, symptom burden, and inconsistent clinical messaging were recurrently identified as key determinants of poor adherence [60, 64]. Educational interventions led by dedicated renal dietitians, including stage-based education and multicomponent approaches combining nutritional counselling and psychological support, were associated with improved dietary compliance and biochemical outcomes [62, 63].

Qualitative studies provided complementary insights into patient and caregiver experiences [65, 66]. Excessive dietary restrictions, unclear or conflicting information, emotional burden, and limited patient involvement in decision-making emerged as major barriers, whereas empathetic communication, tailored education, cultural adaptation, and multidisciplinary coordination were perceived as facilitators of adherence and satisfaction with care.

Observational evidence further underscored practical challenges to adherence, including fatigue, difficulties understanding dietary advice, and limited ability to maintain preferred eating patterns, reinforcing the need for clearer and more individualized nutritional guidance [67].

Five studies [39–41, 68, 69] consistently underscored the relevance of personalized nutritional approaches for improving dietary adherence and clinical outcomes in HD. Two studies specifically highlighted the need to tailor interventions to patients’ clinical, cultural, and functional characteristics, incorporating strategies such as targeted supplementation, psychological support, and ongoing monitoring of PEW [40, 69]. Conceptual models advocating greater dietary flexibility were also proposed, including an ‘aspirational diet’ that prioritized balanced, enjoyable, food-based patterns and allowed the adjunctive use of chelating agents to support metabolic control without compromising safety [41]. Similarly, patient-centred frameworks integrating individual preferences, food culture, and dietary flexibility into therapeutic planning were described [39], alongside calls to revise traditional restrictive recommendations in favour of more varied and sustainable dietary patterns that include vegetables, healthy fats, and high-quality protein sources [68]. In this context, high-quality protein was conceptualized not only in terms of essential amino acid composition and biological value, but also with regard to bioavailability, phosphorus burden, and degree of processing. Accordingly, personalized strategies incorporated selected animal-based proteins as well as minimally processed plant-based sources, while limiting ultraprocessed products containing phosphate additives [39, 41, 68].

### Advanced nutritional assessment and considerations in older adults undergoing HD

Six studies [70–75] examined morphofunctional assessment tools and nutritional status in adults undergoing HD, including older individuals (Table 3). Overall, the evidence suggested an increasing use of objective and noninvasive methods, such as muscle ultrasound and bioelectrical impedance analysis (BIA), compared with traditional biochemical indicators.

Muscle ultrasound was consistently validated as a reliable tool for assessing muscle mass and sarcopenia, showing strong correlations with dual-energy X-ray absorptiometry (DXA), BIA, and functional measures, and clinically relevant associations with hospitalization, mortality, and frailty risk [71, 72]. Multifrequency BIA (MF-BIA) was also highlighted as a practical method to estimate lean mass and hydration status, particularly when combined with MIS and muscle ultrasound as part of an integrated morphofunctional screening approach [73].

In older patients undergoing HD, several studies reported that traditional parameters such as BMI and serum albumin underestimate nutritional risk [74, 75]. Reduced protein intake, higher consumption of ultraprocessed foods, loss of lean mass and muscle strength, and a high prevalence of PEW and frailty were consistently described [75]. The MIS emerged as a robust composite marker of nutritional and inflammatory risk, with higher scores were independently associated with older age, lower protein intake, and longer dialysis vintage [70].

### Overall synthesis of results

There was substantial heterogeneity in nutritional interventions, assessment tools, and reported outcomes across the included studies. Personalized nutritional strategies, particularly when combined with structured education, psychosocial support, and patient-centred flexibility, were associated with improvements in nutritional markers, dietary adherence, and selected functional outcomes. In particular, some studies incorporated performance-based assessments, such as handgrip dynamometry and the Short Physical Performance Battery, alongside muscle evaluation using BIA and ultrasonography. However, these more comprehensive approaches were reported in only a minority of studies. Advanced morphofunctional tools were associated with improved detection of nutritional risk beyond conventional biochemical parameters, particularly in older adult patients undergoing HD.

Methodological variability, short intervention durations, and inconsistent outcome reporting limited cross-study comparability and precluded the formulation of standardized recommendations. This diversity across interventions and assessment strategies suggests the absence of a single dominant care model and supports the need for adaptable, context-sensitive nutritional pathways rather than uniform protocols.

**Figure 3:**
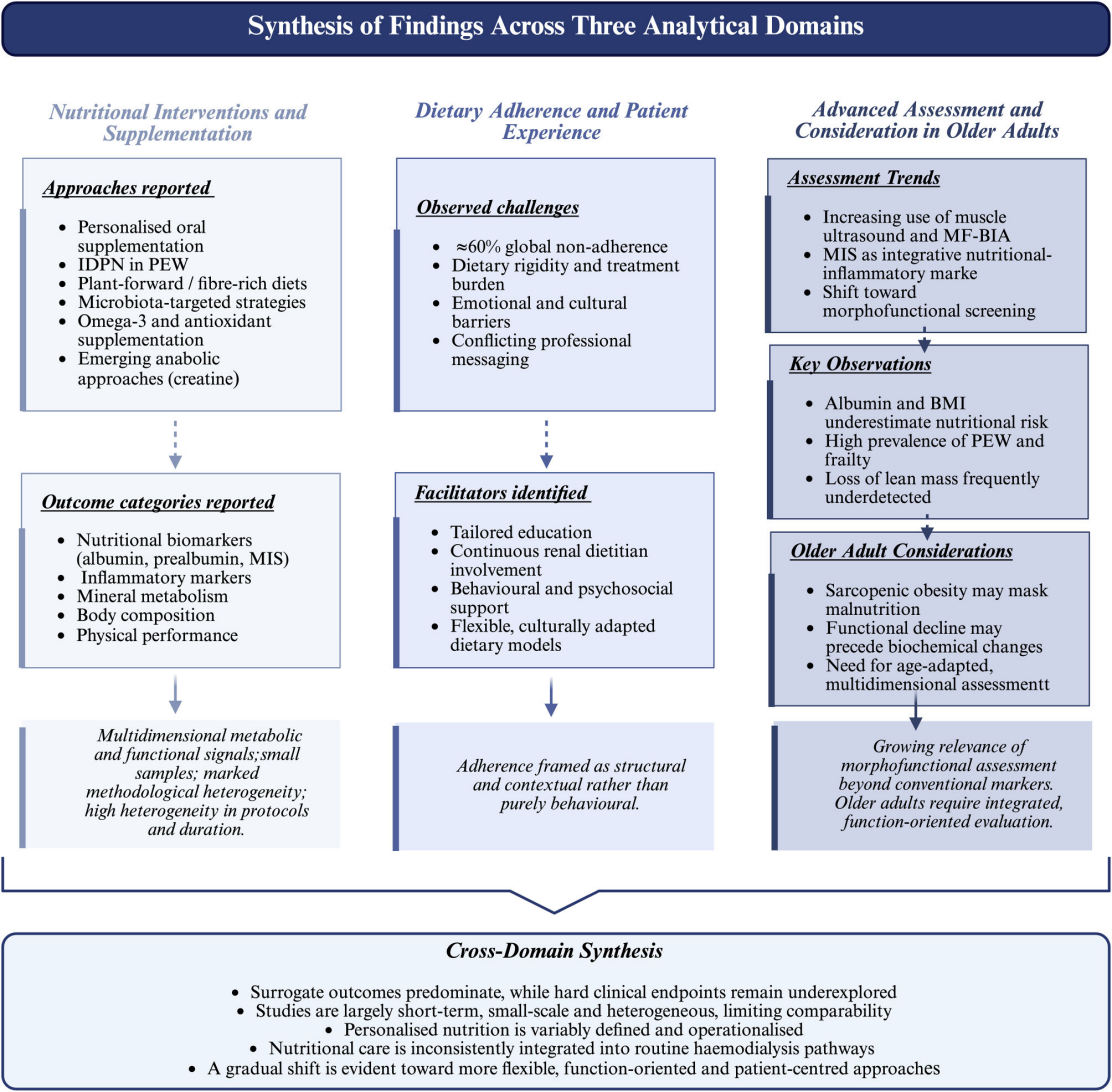
Evidence landscape of nutritional management in HD (2015–2025). Schematic synthesis of findings derived from 30 studies included in this scoping review, organized across three analytical domains: (i) nutritional interventions and supplementation strategies, (ii) dietary adherence and patient experience, and (iii) advanced nutritional assessment, including considerations for older adults undergoing HD. The figure summarizes reported intervention types, outcome categories, and overarching patterns identified across studies. It highlights the predominance of surrogate outcomes, the methodological heterogeneity, and short-term nature of much of the available evidence, as well as the evolving shift from restrictive, laboratory-driven approaches towards more flexible, function-oriented, and patient-centred models of care. Created by the authors using BioRender.com.

To provide an integrated overview of the evidence mapped across the three analytical domains, the main reported intervention types, outcome categories, and cross-domain patterns are summarized in Fig. 3. This visual synthesis highlights the multidimensional nature of personalized nutritional care in HD, the predominance of surrogate outcomes, and the methodological and implementation gaps identified across the literature.

To enhance interpretative clarity, outcomes were further considered according to their level of clinical translation, including hard clinical endpoints, surrogate or intermediate markers, and patient-reported outcomes (Supplementary Table S6). Hard outcomes reflected broader disease trajectory (e.g. hospitalization, dialysis tolerance, and prevalence of PEW), whereas surrogate markers encompassed biochemical and morphofunctional indicators of nutritional status. Patient-reported outcomes captured experiential dimensions such as quality of life and dietary adherence.

## DISCUSSION

To our knowledge, this scoping review provides the first integrative synthesis of clinical barriers, nutritional strategies, and implementation-related considerations in the nutritional management of patients undergoing HD, drawing on evidence from 30 studies. Nutritional care in HD represents a complex and multifactorial component of routine clinical practice, influenced by metabolic derangements, chronic inflammation, psychosocial burden, and organizational constraints intrinsic to RRT. The findings highlight substantial heterogeneity in nutritional assessment methods, intervention strategies, and models of care, reflecting both variability in clinical practice and persistent gaps in implementation.

**Figure 4:**
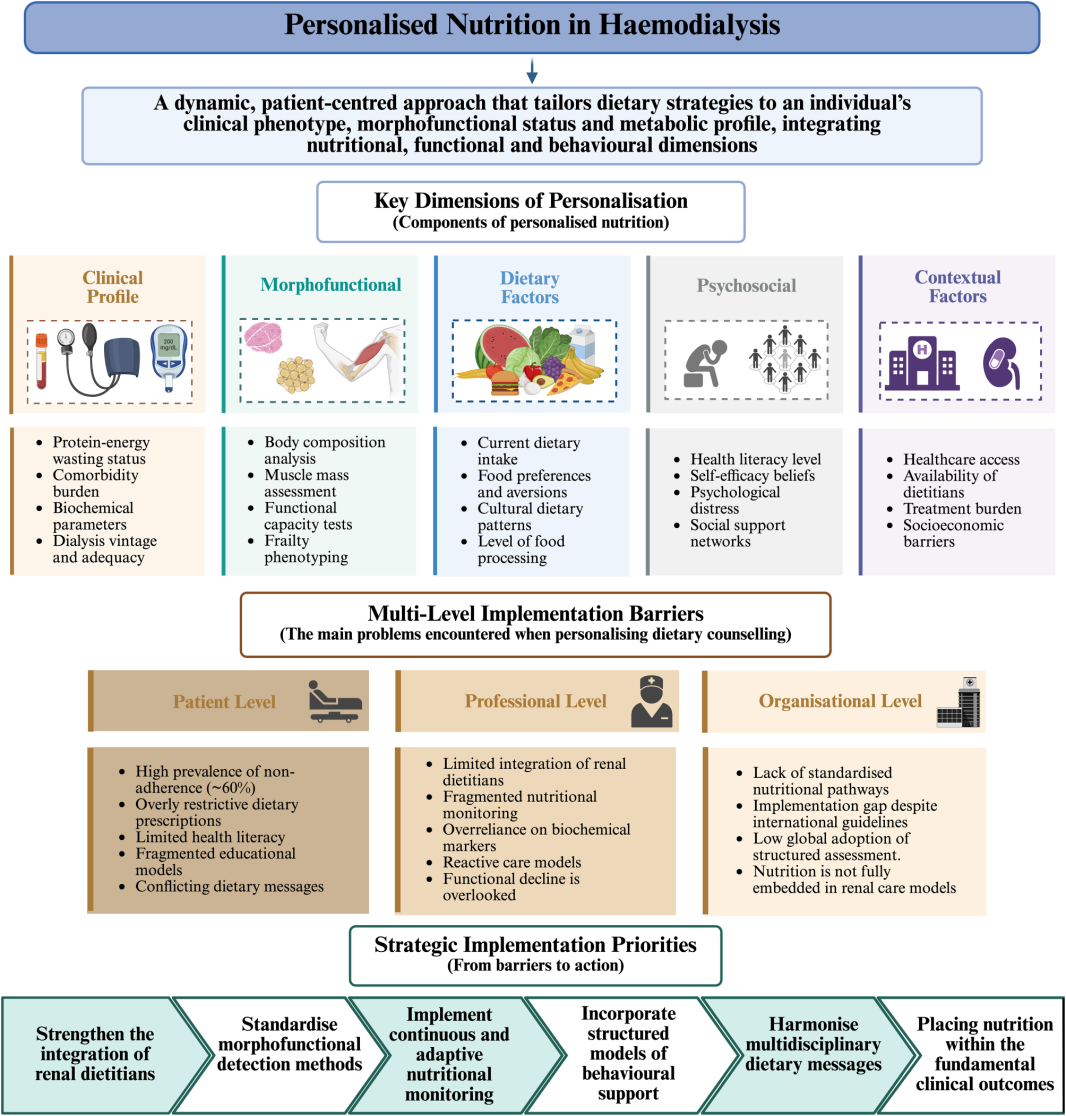
Conceptual framework of personalized nutrition in HD and multilevel implementation barriers. Conceptual framework derived from the synthesis of the included studies (2015–2025). The upper section illustrates the core dimensions of personalized nutrition in HD, integrating clinical phenotype, morphofunctional status, dietary factors, psychosocial determinants, and contextual elements within a patient-centred model. The middle section summarizes multilevel implementation barriers identified across the literature (patient, professional, and organizational levels). The lower section outlines strategic implementation priorities aimed at translating personalized nutritional care into routine clinical practice. Created by the authors using BioRender.com.

Overall, the mapped evidence reinforces the central role of nutrition within patient-centred, multidisciplinary HD care, with close links to functional status, treatment tolerance, and quality of life. However, the concept of personalized nutrition is applied inconsistently across the literature, ranging from isolated dietary modifications or supplement prescriptions to more comprehensive, multimodal approaches integrating morphofunctional assessment, patient preferences, and behavioural support. This variability in how ‘personalization’ is defined and operationalized limits conceptual coherence and hampers direct comparison across studies. It also contributes to implementation gaps, as the absence of a shared framework makes translation into routine clinical practice more challenging. Based on the synthesis of evidence across the three analytical domains, we therefore propose an integrative conceptual framework that clarifies the core dimensions of personalized nutrition in HD and delineates the multilevel barriers influencing its implementation in real-world settings (Fig. 4).

### Barriers to addressing nutrition in patients undergoing HD

This review indicates that barriers to effective nutritional management in HD are not isolated or transient but represent a persistent, system-level challenge spanning individual behaviours, professional practice, and organizational structures. Importantly, these barriers help explain the enduring gap between the recognized clinical relevance of nutrition in HD and its limited, inconsistent integration into routine care across settings.

At the individual level, poor dietary adherence affecting ∼60% of patients emerges as a structural rather than purely behavioural issue [60, 61]. The literature consistently suggests that nonadherence is less a consequence of patient unwillingness and more a reflection of dietary prescriptions perceived as overly restrictive, culturally misaligned and difficult to reconcile with everyday life [64, 65, 67]. The association between dietary rigidity, higher MIS scores, and poorer quality of life underscores how nutritional failure in HD is tightly interwoven with functional decline, fatigue, and treatment burden, rather than isolated dietary choices [68, 76]. From this perspective, nonadherence should be interpreted as a marker of broader vulnerability rather than intentional nonadherence.

Poor health literacy and limited understanding of diet–laboratory relationships further compound these difficulties, particularly among older and socially vulnerable patients [65, 66]. While improved nutritional knowledge has been associated with better adherence [65], the evidence also reveals a recurring disconnect between knowledge acquisition and sustained behavioural change [77, 78]. This gap highlights a key limitation of education-centred models when delivered in isolation, without motivational support, continuity, and structured follow-up, or when not integrated into routine HD care pathways [79, 80]. Moreover, inconsistent or conflicting dietary messages from healthcare professionals appear to undermine trust and engagement, suggesting that the problem is not solely patient-related but embedded within fragmented care delivery [66, 81].

At a professional level, the limited integration of renal dietitians within HD units is repeatedly identified as a discrepancy between evidence-informed recommendations and routine practice [34, 42, 68]. Only a minority of studies reported the systematic involvement of renal dietitians in patient follow-up. This is frequently associated with intermittent nutritional assessment and an excessive reliance on indicators such as serum albumin or BMI, which are widely described as underestimating the true risk of malnutrition [64, 73]. Despite its widespread use in clinical practice, serum albumin should not be interpreted as a direct indicator of nutritional intake in patients undergoing HD, as inflammation, vascular permeability, fluid shifts, and comorbidity may substantially influence albumin concentrations independently of dietary protein consumption. Consequently, low albumin levels can reflect inflammatory or catabolic processes rather than isolated nutritional deficiency [82, 83]. These constraints limit the development of sustained interventions, continuous education, and dynamic dietary prescription adjustments, and favour reactive rather than preventive approaches to nutritional care [84].

Global data revealed that only 36% of centres employed permanent renal dietitians, and that clinical nutrition was not formally recognized as a regulated discipline within nephrology in >40% of countries [85]. This systemic deficit may further hinder the integration of nutrition into renal care, particularly in settings with limited resources, where monitoring tends to focus on biochemistry rather than function.

At an organizational level, the absence of standardized nutritional screening and monitoring protocols is frequently described as reinforcing care models focused primarily on biochemical correction rather than on functional preservation and quality of life. Although international guidelines such as *Kidney Disease: Improving Global Outcomes* (KDIGO) [42] and the *European Society for Clinical Nutrition and Metabolism* (ESPEN) [43] outline principles for periodic nutritional assessment, most reviewed studies report irregular or absent implementation in routine practice [20, 40, 65, 66, 73]. Similarly, global survey data indicate that <25% of HD centres have implemented standardized nutritional assessment protocols [85]. This lack of systematization is commonly discussed as a contextual factor contributing to why nutrition is often approached as a complementary component of care, despite growing research interest in its relationship with survival, frailty, and cardiovascular outcomes [86–88].

### Clinical opportunities, courses of action, and improvement

#### Reinforcing the role of the renal dietitian and multidisciplinary care

The evidence suggests the importance of reinforcing the role of the renal dietitian as a key member of the multidisciplinary team in HD units. Their systematic involvement is described as enabling the implementation of personalized nutritional interventions based on regular assessments and the progressive adjustment of dietary prescriptions [40, 63, 67, 84, 85]. There is a need to develop standardized protocols that incorporate the nutrition team from the early stages of RRT to ensure consistency and long-term effectiveness [40, 53, 63].

#### Structured nutrition education and behavioural support

This scoping review identified the development of structured nutrition education programmes targeting both patients and caregivers as a key priority [39, 65, 68]. These initiatives should promote practical skills, self-efficacy, and behavioural change while addressing daily barriers and emotional challenges [67]. To maximize their impact, the programmes must be adapted to varying levels of health literacy and sociocultural backgrounds and delivered through diverse, patient-centred formats, including individual counselling, group workshops, and digital tools [66]. In this context, telehealth resources such as mobile applications and remote monitoring platforms have been investigated as additional educational tools. These have been associated with improved nutritional parameters and high patient acceptability [89, 90]. However, their effectiveness depends on accessibility, usability, and clinical validation, and when used to complement face-to-face nutritional care, digital tools remain limited by digital illiteracy, age-related impairments, and unequal access to technology, which may widen health disparities [91]. Furthermore, integrating motivational and psychoeducational approaches such as motivational interviewing, realistic goal setting, and shared decision-making has been suggested as a way to improve adherence, empower patients, and encourage sustained engagement with nutritional self-management [64, 67, 69].

#### Targeted nutritional interventions in selected clinical contexts

Beyond educational and organizational strategies, selected advanced nutritional interventions may play a role in the comprehensive management of patients undergoing HD. IDPN has been described as an effective option for improving nitrogen balance and markers of protein status in patients with PEW or inadequate oral intake [36, 52, 69]. However, its use requires careful patient selection, multidisciplinary coordination, and close metabolic monitoring to ensure safety [34]. Importantly, the available evidence is limited by small sample sizes, short intervention durations, and the absence of hard clinical endpoints such as hospitalization, quality of life, or mortality [92, 93]. In addition, heterogeneity in formula composition, dosing, and treatment duration limits comparability and precludes robust recommendations. Accordingly, IDPN should be considered a complementary strategy restricted to selected patient subgroups within structured clinical protocols [32, 34].

Individualized oral supplementation represents a more widely applicable approach to meeting nutritional requirements, particularly when protein- or amino-acid-enriched formulations are tailored to metabolic demands, nutritional status, and gastrointestinal tolerance [40, 50, 51]. This aligns with current KDIGO and ESPEN recommendations, which conceptualize PEW as a modifiable condition driven by inflammation and metabolic stress rather than an inevitable consequence of kidney failure.

Nevertheless, methodological limitations remain a consistent feature of the literature. Most studies are characterized by small cohorts, short follow-up periods, limited incorporation of objective functional outcomes, and heterogeneity in intervention design, as well as potential sources of bias. While the overall direction of the effect indicates potential benefits with regard to protein metabolism and inflammatory markers, higher-quality, longer-term studies are required in order to determine the clinical effectiveness and optimal implementation [94].

Emerging strategies such as creatine monohydrate supplementation have also been explored in HD populations. Preliminary data suggest potential benefits on muscle mass and functional performance without major safety concerns [59], supported by experimental and mechanistic rationale related to muscle energetics in CKD [95, 96]. However, current evidence remains scarce and inconsistent, and creatine should therefore be regarded as an exploratory intervention rather than an established therapeutic option.

#### From the ‘universal renal diet’ to flexible, binder-supported models and microbiota-targeted strategies

The findings of this review support a shift away from the traditional concept of a ‘universal renal diet’, which has historically been characterized by rigid and poorly individualized restrictions. Instead, we should embrace more flexible dietary models that prioritize variety, cultural appropriateness, and patient acceptability, while still ensuring metabolic safety [39, 41, 68]. This evolving approach increasingly considers food processing, nutrient bioavailability, and individual risk profiles. In this context, evidence indicating higher bioavailability of potassium and phosphorus from food additives compared with naturally occurring sources may partly explain the limited effectiveness of blanket dietary restrictions [97]. The rational use of phosphate and potassium binders has therefore been discussed as a means to safely expand dietary options, particularly by limiting exposure to inorganic phosphate while maintaining mineral control and supporting diet quality and quality of life [42, 68, 98].

In parallel, modulation of the gut microbiota through prebiotics, probiotics, and symbiotics has been explored as a complementary nutritional strategy in HD. Available evidence suggests potential effects on uremic toxin production, systemic inflammation, and intestinal homeostasis, with possible downstream benefits for nutritional status and protein metabolism [55, 56]. A small body of evidence also suggests that combining nutritional supplementation with probiotics may influence patient-reported outcomes, including psychological wellbeing and quality of life [99]. However, clinical translation remains limited by small sample sizes, heterogeneity in formulations and dosing, and the absence of standardized outcome measures. Accordingly, microbiota-targeted interventions should currently be regarded as adjunctive and exploratory, pending further standardization and integration into comprehensive nutritional care models [100, 101].

Finally, increasing attention has been directed towards omega-3 fatty acids and antioxidant-based interventions as strategies targeting oxidative stress, mitochondrial dysfunction and chronic inflammation, mechanisms closely linked to frailty, sarcopenia, and PEW [57, 58]. Within the mapped evidence, large randomized trials have reported associations between omega-3 supplementation and cardiovascular outcomes in HD populations [102]. Collectively, these developments reflect a broader shift towards investigating nutritional interventions not only for metabolic control, but also for their potential impact on clinically meaningful outcomes within integrated renal care frameworks.

#### Plant-based dietary patterns and culturally adapted flexible diets

In recent years, there has been an increasing focus in the scientific literature on the role of plant-based foods in the diets of patients undergoing HD, which have traditionally been restricted due to concerns regarding hyperkalaemia [103]. Emerging studies suggest that the implementation of well-planned and professionally supervised plant-based dietary patterns is feasible in this population and has been associated with improvements in overall diet quality, higher intakes of fibre and antioxidant micronutrients, and adequate adherence to established protein and energy requirements [103, 104]. Across the included studies, plant-centred dietary approaches implemented under individualized nutritional supervision were generally reported to be compatible with stable serum potassium levels, while some studies also described favourable changes in phosphorus metabolism, FGF-23 concentrations, dry weight control, inflammatory markers, and nutritional status [53, 54].

Overall, the reviewed literature reflects a growing interest in flexible and culturally adapted dietary approaches in HD care. Individualized nutrition education is repeatedly identified as an important contextual factor in facilitating the integration of plant-based foods and is commonly discussed in relation to adherence, patient satisfaction, and maintenance of metabolic control [105, 106].

#### Nutritional vulnerability of older adults undergoing HD

Conversely, older adult patients undergoing HD constitute a particularly vulnerable group from a nutritional perspective, owing to the convergence of multiple physiological, functional, and social factors that compromise both nutrient intake and utilization [74]. The evidence presented in this review indicates that the dietary quality of this population is often suboptimal, characterized by a high reliance on ultraprocessed foods and poor adherence to healthy dietary patterns [74, 75]. This nutritional deterioration is commonly described in relation to uremic anorexia, alterations in taste and smell, fatigue, functional limitations in food preparation, and loss of autonomy, together with social and familial barriers that reduce daily dietary support. These interrelated factors are consistently associated with a sustained state of nutritional vulnerability and an increased risk of PEW, sarcopenia, and frailty in older patients undergoing HD [74, 107].

Furthermore, traditional methods of nutritional assessment, such as BMI, serum albumin, or creatinine, appear to have limited sensitivity in older adult patients undergoing HD, as they do not fully capture age-related physiological changes or the characteristic redistribution of body composition observed in this population [73]. Multiple studies have reported these limitations [107–110], and the reviewed literature increasingly discusses the relevance of age-appropriate clinical criteria and the use of more comprehensive morphofunctional assessment approaches. These approaches integrate measures of body composition, muscle strength, and inflammatory status and are described as being better aligned with the clinical, functional, and cognitive particularities of older adult patients undergoing HD [73, 74].

#### The role of morphofunctional assessment in HD care

The evidence presented in this review aligns closely with the principles outlined in major international clinical guidelines, such as those from Kidney Disease Outcomes Quality Initiative (KDOQI) [34] and KDIGO [42], which underscore the significance of systematic and structured nutritional assessment in patients with ESRD undergoing HD. In the reviewed literature, there is a frequent discussion of validated tools including the Subjective Global Assessment and the MIS. This reflects the recognition that no single assessment method provides sufficient sensitivity and specificity for identifying PEW. In this context, several studies report that MIS is associated not only with nutritional status but also with inflammatory markers, hospitalization risk, and mortality among patients undergoing HD [70, 73].

Likewise, the reviewed literature increasingly describes the incorporation of complementary morphofunctional assessments using objective techniques, such as MF-BIA, muscle ultrasonography, and handgrip dynamometry, as approaches that may enhance the individualized evaluation of nutritional and functional status in patients undergoing HD. These techniques are also discussed in relation to improved assessment of fluid status, with potential implications for volume management and ultrafiltration planning [111]. In this context, international clinical guidelines, including those from KDOQI [34], acknowledge the use of MF-BIA, preferably performed at least 30 min after the HD session, and recognize DXA as a reference method, while also noting its susceptibility to hydration-related variability. Consistent with this, studies included in this review [71, 72] report that quadriceps muscle ultrasonography is an accessible and reproducible method that shows correlations with DXA, BIA-derived parameters, and clinically relevant outcomes, supporting its consideration for the early identification of sarcopenia in patients undergoing HD [112].

Finally, the reviewed literature increasingly frames morphofunctional assessment within broader patient-centred care models, in which nutritional management is positioned as an integral component of routine HD practice, supported by professional training, effective communication strategies, and interdisciplinary collaboration [39, 69].

This scoping review has several limitations. First, despite implementing a structured and transparent search strategy, restricting the search to four electronic databases and English-language publications may have resulted in the omission of relevant studies. Second, the review focused on studies published between 2015 and 2025 in order to capture contemporary evidence reflecting recent paradigm shifts in personalized nutrition. While this approach enabled detailed mapping of current practice and emerging strategies, earlier foundational literature that shaped the development of nutritional management in HD was not included. Thirdly, substantial methodological heterogeneity was observed across the included studies in terms of design, interventions, populations, and reported outcomes. This limited comparability and precluded quantitative synthesis. Additionally, much of the available evidence consisted of review articles and small-scale or short-term primary studies, particularly in the interventional domain. Many studies emphasized intermediate markers, whereas long-term, patient-centred, and hard clinical outcomes were examined less frequently. These limitations are consistent with the exploratory nature of scoping reviews and highlight the need for more standardized, longitudinal, implementation-oriented primary research in HD nutrition.

Despite these limitations, this scoping review provides an integrative, multidimensional overview of nutritional management in HD, considering biomedical, psychosocial, educational, and organizational factors. Including both quantitative and qualitative evidence enabled objective barriers to be identified, such as malnutrition, lack of standardized protocols, and limited resources, as well as subjective factors related to dietary perception, motivation, and emotional support. This review’s applied, practice-oriented perspective supports the identification of strategies to enhance nutritional care at different levels, including integrating renal dietitians into nephrology teams and moving towards more coordinated, personalized, and patient-centred models of care. In addition, this review proposes a structured conceptual definition of personalized nutrition in HD, synthesising its clinical, behavioural, and organizational components into a coherent framework to guide both research and practice.

### Future directions and clinical implications

This scoping review highlights the ongoing discrepancy between nutritional research findings and their incorporation into standard HD care. Despite the existence of robust associations between nutritional status, inflammation, functional decline, and patient-centred outcomes, the implementation of nutritional strategies remains inconsistent and is largely constrained by professional and organizational factors.

There is an increasing body of literature supporting a shift towards flexible, personalized, function-oriented nutritional approaches. However, advancing this transition will require pragmatic, implementation-focused research capable of addressing real-world constraints and supporting sustainable nutritional care models in HD. From a clinical perspective, these findings suggest that renal dietitians and morphofunctional assessment should be integrated into routine HD care to enable effective personalized nutrition.

To further clarify research priorities emerging from this scoping review, reported outcome domains were contrasted with areas that remain comparatively underexplored in the current literature (Table 4). This comparative mapping provides a structured overview of the current evidence landscape and highlights key directions for future investigation.

**Table 4:** Outcome reporting patterns and future research priorities in nutritional management of patients undergoing HD.

Outcome domain	Commonly reported	Comparatively underexplored	Future research implications
Biochemical and inflammatory markers	Serum albuminPrealbuminC-reactive proteinPhosphorus parametersPotassiumNormalized protein catabolic rate	Micronutrient panels (vitamins B, C, D, E)Trace elements (zinc, selenium)Uraemic toxins (indoxyl sulphate, p-cresyl sulphate)Advanced inflammatory cytokines (TNF-α, IL-1β)Integration with longitudinal clinical endpoints	Link multimarker panels to hard outcomes (mortality, hospitalization) through adequately powered, long-term cohort studies; establish HD-specific reference ranges
Dietary intake and adherence	Total energy intakeTotal protein intakeGeneral dietary patternsNonvalidated adherence questionnaires	Diet quality indices (HEI, Mediterranean, DASH scores)Ultraprocessed food consumptionFood additive exposure (phosphorus, potassium)Nutrient timing and distributionLong-term dietary sustainabilityValidated adherence scales	Incorporate food processing classification, nutrient bioavailability and behavioural frameworks; develop and validate HD-specific dietary adherence tools; assess long-term sustainability
Body composition	Lean mass (BIA)BMIMid-arm circumferenceMuscle thickness via ultrasound (emerging)	Sarcopenic obesity phenotypingVisceral adiposity quantificationPhase angle (from BIA)Standardized longitudinal muscle trajectory dataDXA in patients undergoing HD	Establish standardized protocols for prospective monitoring of sarcopenia progression; validate phase angle cutoffs; integrate body composition into routine nutritional assessment
Morphofunctional assessment tools	MISSubjective Global AssessmentMuscle ultrasound (selected studies)	Routine integration into clinical care pathwaysCombined morphofunctional indicesAge-adapted assessment criteria for older adult patientsGeriatric Nutritional Risk Index implementation	Development of structured, feasible screening algorithms for routine HD units; training programmes for nonspecialist staff; validation in diverse settings
Functional capacity and performance	Handgrip strength (inconsistently reported)Short Physical Performance Battery (limited studies)	Comprehensive frailty assessment (Fried, FRAIL scale)Gait speed, Timed Up, and GoActivities of daily living scalesLong-term functional decline trajectoriesFrailty transitions over time	Incorporate performance-based and patient-relevant functional endpoints into nutritional intervention trials; monitor trajectories rather than isolated time points
Patient-reported outcomes	Quality of life (generic tools, sporadic)Treatment satisfaction (rare)	Symptom burden (fatigue, anorexia, gastrointestinal)Dietary quality of lifeSleep qualityDepression and anxiety screeningSelf-efficacy and empowermentFood satisfaction and dietary flexibility	Systematically integrate validated, disease-specific PRO measures; establish minimal clinically important differences; explore associations with adherence and survival
Hard clinical outcomes	PEW prevalenceDialysis tolerance (select reports)Short-term biochemical changes	All-cause and cardiovascular mortalityHospitalization rates and length of stayCardiovascular events (MI, stroke, heart failure)Infection ratesTime-to-event analyses	Conduct adequately powered, multicentre interventional trials with sufficient follow-up to detect clinically meaningful differences in patient-important outcomes
Gut microbiota and metabolomics	Short-chain fatty acids (limited)Selected uraemic toxins	Comprehensive microbiome profiling (16S rRNA, metagenomics)Metabolomic signaturesGut-kidney axis biomarkersMicrobiota-targeted intervention mechanisms	Mechanistic studies linking microbiota changes to clinical outcomes; evaluate feasibility and sustainability of microbiota-modulating interventions in routine care
Implementation factors and health economics	Reported barriers to dietary adherenceRenal dietitian involvement (descriptive)	Cost-effectiveness analysesBudget impact modelsIntervention acceptability and feasibilitySustainability in resource-limited settingsHealth equity and access disparitiesScalable personalized nutrition models	Implementation science studies addressing real-world translation barriers; economic evaluations to inform policy and reimbursement; equity-focused research in underserved populations

BIA: bioelectrical impedance analysis; BMI: body mass index; CRP: C-reactive protein; DASH: dietary approaches to stop hypertension; DXA: dual-energy X-ray absorptiometry; HD: haemodialysis; HEI: healthy eating index; IL: interleukin; MI: myocardial infarction; MIS: malnutrition–inflammation score; PEW: protein-energy wasting; PRO: patient-reported outcome; TNF-α: tumor necrosis factor-alpha.

## CONCLUSIONS

This scoping review provides an overview of the nutritional strategies, assessment tools, and contextual influences described in HD literature. The mapped evidence shows substantial variability across studies and settings, reflecting the complexity of nutritional care for this patient group. Rather than indicating a single dominant model, the literature suggests multiple approaches, which are shaped by patient characteristics, professional practices, and local resources. By synthesizing these patterns, this review identifies areas in which the field is evolving and highlights dimensions that warrant further exploration. Future studies could benefit from designs that clarify how different nutritional strategies operate in different contexts, and how they can be meaningfully integrated into routine care. These findings support the need to further conceptualize and operationalize personalized nutrition in HD, ensuring that future research and clinical pathways are aligned with patient-centred and context-responsive care models.

## Supplementary Material

sfag117_Supplemental_File

## Data Availability

Not applicable.
