# Supplementary material for: Personalized nutrition in haemodialysis: a scoping review of studies published between 2015 and 2025
Source: Clin Kidney J. 2026 Apr 16;19(5):sfag117. doi: 10.1093/ckj/sfag117 (PMC13142157; doi:10.1093/ckj/sfag117)
Supplement: sfag117_Supplemental_File [file sfag117_supplemental_file.docx]

**Supplementary information of contents**

**Supplementary Table S1:** PRISMA-ScR Checklist

**Supplementary information 1:** Search strategy

**Supplementary Table S2:** Electronic search strategies used in the scoping review

**Supplementary information 2:** Consideration of methodological quality

**Supplementary Table S3:** Methodological characteristics of studies included in the scoping review (n=30)

**Supplementary Table S4:** Population characteristics

**Supplementary Table S5:** Geographic distribution of included studies

**Supplementary Table S6:** Mapping of outcomes assessed across analytical domains in included studies (n=30)

**Supplementary Table S1. PRISMA-ScR Checklist**

| **SECTION** | **ITEM** | **PRISMA-ScR CHECKLIST ITEM** | **REPORTED ON PAGE #** |
| --- | --- | --- | --- |
| **TITLE** | | | |
| Title | 1 | Identify the report as a scoping review. | Title page |
| **ABSTRACT** | | | |
| Structured summary | 2 | Provide a structured summary that includes (as applicable): background, objectives, eligibility criteria, sources of evidence, charting methods, results, and conclusions that relate to the review questions and objectives. | Abstract |
| **INTRODUCTION** | | | |
| Rationale | 3 | Describe the rationale for the review in the context of what is already known. Explain why the review questions/objectives lend themselves to a scoping review approach. | Introduction |
| Objectives | 4 | Provide an explicit statement of the questions and objectives being addressed with reference to their key elements (e.g., population or participants, concepts, and context) or other relevant key elements used to conceptualize the review questions and/or objectives. | Introduction (last paragraph) |
| **METHODS** | | | |
| Protocol and registration | 5 | Indicate whether a review protocol exists; state if and where it can be accessed (e.g., a Web address); and if available, provide registration information, including the registration number. | Methods – first paragraph |
| Eligibility criteria | 6 | Specify characteristics of the sources of evidence used as eligibility criteria (e.g., years considered, language, and publication status), and provide a rationale. | Methods – Eligibility Criteria |
| Information sources* | 7 | Describe all information sources in the search (e.g., databases with dates of coverage and contact with authors to identify additional sources), as well as the date the most recent search was executed. | Methods – Information Sources |
| Search | 8 | Present the full electronic search strategy for at least 1 database, including any limits used, such that it could be repeated. | Methods; Supplementary Table S1 |
| Selection of sources of evidence† | 9 | State the process for selecting sources of evidence (i.e., screening and eligibility) included in the scoping review. | Methods – Study Selection |
| Data charting process‡ | 10 | Describe the methods of charting data from the included sources of evidence (e.g., calibrated forms or forms that have been tested by the team before their use, and whether data charting was done independently or in duplicate) and any processes for obtaining and confirming data from investigators. | Methods – Data Charting and Synthesis |
| Data items | 11 | List and define all variables for which data were sought and any assumptions and simplifications made. | Methods – Data Charting |
| Critical appraisal of individual sources of evidence§ | 12 | If done, provide a rationale for conducting a critical appraisal of included sources of evidence; describe the methods used and how this information was used in any data synthesis (if appropriate). | Methods – Data Charting and Synthesis; Supplementary Methods |
| Synthesis of results | 13 | Describe the methods of handling and summarizing the data that were charted. | Methods – Data Charting and Synthesis |
| **RESULTS** | | | |
| Selection of sources of evidence | 14 | Give numbers of sources of evidence screened, assessed for eligibility, and included in the review, with reasons for exclusions at each stage, ideally using a flow diagram. | Results; Figure 2 |
| Characteristics of sources of evidence | 15 | For each source of evidence, present characteristics for which data were charted and provide the citations. | Results – Characteristics; Tables 1–3 |
| Critical appraisal within sources of evidence | 16 | If done, present data on critical appraisal of included sources of evidence (see item 12). | Not applicable (explained in Methods & Supplementary) |
| Results of individual sources of evidence | 17 | For each included source of evidence, present the relevant data that were charted that relate to the review questions and objectives. | Results; Tables 1–3 |
| Synthesis of results | 18 | Summarize and/or present the charting results as they relate to the review questions and objectives. | Results – Overall synthesis |
| **DISCUSSION** | | | |
| Summary of evidence | 19 | Summarize the main results (including an overview of concepts, themes, and types of evidence available), link to the review questions and objectives, and consider the relevance to key groups. | Discussion |
| Limitations | 20 | Discuss the limitations of the scoping review process. | Discussion – Limitations |
| Conclusions | 21 | Provide a general interpretation of the results with respect to the review questions and objectives, as well as potential implications and/or next steps. | Conclusions |
| **FUNDING** | | | |
| Funding | 22 | Describe sources of funding for the included sources of evidence, as well as sources of funding for the scoping review. Describe the role of the funders of the scoping review. | Funding section |

JBI = Joanna Briggs Institute; PRISMA-ScR = Preferred Reporting Items for Systematic reviews and Meta-Analyses extension for Scoping Reviews.

* Where *sources of evidence* (see second footnote) are compiled from, such as bibliographic databases, social media platforms, and Web sites.

† A more inclusive/heterogeneous term used to account for the different types of evidence or data sources (e.g., quantitative and/or qualitative research, expert opinion, and policy documents) that may be eligible in a scoping review as opposed to only studies. This is not to be confused with *information sources* (see first footnote).

‡ The frameworks by Arksey and O’Malley (6) and Levac and colleagues (7) and the JBI guidance (4, 5) refer to the process of data extraction in a scoping review as data charting*.*

§ The process of systematically examining research evidence to assess its validity, results, and relevance before using it to inform a decision. This term is used for items 12 and 19 instead of "risk of bias" (which is more applicable to systematic reviews of interventions) to include and acknowledge the various sources of evidence that may be used in a scoping review (e.g., quantitative and/or qualitative research, expert opinion, and policy document). *From:* Tricco AC, Lillie E, Zarin W, O'Brien KK, Colquhoun H, Levac D, et al. PRISMA Extension for Scoping Reviews (PRISMAScR): Checklist and Explanation. Ann Intern Med. 2018;169:467–473. [doi: 10.7326/M18-0850](http://annals.org/aim/fullarticle/2700389/prisma-extension-scoping-reviews-prisma-scr-checklist-explanation).

**Supplementary information 1. Search strategy**

A comprehensive literature search was conducted on August 10, 2025, using four major electronic databases: PubMed/MEDLINE, Scopus, Web of Science, and Europe PMC, selected for their broad coverage of biomedical, nephrology, and clinical nutrition research.

The search strategy was initially developed for PubMed/MEDLINE using a combination of Medical Subject Headings (MeSH) and free-text keywords and subsequently adapted to the syntax and indexing systems of the remaining databases. Search terms included combinations of the following keywords: “hemodialysis,” “haemodialysis,” “renal dialysis,” “nutrition therapy,” “nutritional management,” “diet therapy,” “dietary intervention,” “chronic kidney disease,” “renal failure,” and “malnutrition.” Boolean operators (“AND”, “OR”) were used to refine and optimize retrieval.

The general PubMed equation was:

(“Hemodialysis”[MeSH] OR “Renal Dialysis”[MeSH] OR hemodialysis OR haemodialysis OR “renal dialysis”) AND (“Nutrition Therapy”[MeSH] OR “nutrition therapy” OR “nutritional management” OR “diet therapy” OR “dietary intervention”) AND (“Renal Insufficiency, Chronic”[MeSH] OR “renal failure” OR “chronic kidney disease” OR malnutrition)

The PubMed/MEDLINE search strategy served as the reference framework and was adapted as necessary to the syntax and indexing characteristics of the remaining databases, with simplifications applied where appropriate to maximise sensitivity while maintaining relevance.

Searches were limited to human studies published in English between January 2015 and August 2025, focused on title, abstract, or keywords. This time frame was selected to capture contemporary evidence reflecting current HD practices, nutritional assessment tools, and intervention strategies.

**Supplementary Table S2. Electronic search strategies used in the scoping review**

| **Database** | **Search strategy** |
| --- | --- |
| **PubMed / MEDLINE** | (“Hemodialysis”[MeSH] OR “Renal Dialysis”[MeSH] OR hemodialysis OR haemodialysis OR “renal dialysis”) AND (“Nutrition Therapy”[MeSH] OR “nutrition therapy” OR “nutritional management” OR “diet therapy” OR “dietary intervention”) AND (“Renal Insufficiency, Chronic”[MeSH] OR “renal failure” OR “chronic kidney disease” OR malnutrition) |
| **Scopus** | TITLE-ABS-KEY ((“hemodialysis” OR “haemodialysis” OR “renal dialysis”) AND (“nutrition therapy” OR “nutritional management” OR “diet therapy” OR “dietary intervention”) AND (“renal failure” OR “chronic kidney disease” OR malnutrition)) |
| **Web of Science** | TI = (hemodialysis OR haemodialysis) AND TS = (nutrition* OR diet*) AND TS = (care OR management OR adherence) |
| **Europe PMC** | (“hemodialysis” OR “haemodialysis”) AND (“diet therapy” OR “dietary intervention”) |

HD, haemodialysis; MeSH, Medical Subject Headings; TI, title; TS, topic; TITLE-ABS-KEY, title, abstract and keywords; CKD, chronic kidney disease.

**Supplementary information 2. Consideration of methodological quality**

In line with the objectives and principles of a scoping review, this work aimed to map, characterise and summarise the available evidence on nutritional management in adult haemodialysis patients, rather than determining the effectiveness of specific interventions or performing quantitative comparisons across studies. In line with current methodological guidance for scoping reviews, no formal quantitative risk-of-bias assessment or numerical quality scoring system was applied.

Instead, the methodological characteristics and reporting features of the included studies were examined descriptively and considered narratively. Attention was paid to study design, population characteristics, intervention complexity, outcome selection, and contextual factors relevant to implementation. This approach was chosen to preserve the exploratory nature of the review while enabling transparent appraisal of the methodological strengths and limitations of heterogeneous study types.

For observational studies, selected methodological aspects were considered in light of the Strengthening the Reporting of Observational Studies in Epidemiology (STROBE) guidelines. These included the clarity of the study objectives and the definition of the study population; the appropriateness of the outcome measures; the handling of confounding factors; and the transparency of statistical reporting.

For randomised and non-randomised interventional studies, key design and reporting features informed by the Consolidated Standards of Reporting Trials (CONSORT) statement were considered, including randomisation procedures (where applicable), description of interventions, follow-up duration, completeness of outcome reporting, and reporting of adverse events.

For systematic, narrative and scoping reviews, general methodological elements drawn from the A Measurement Tool to Assess Systematic Reviews (AMSTAR 2) checklist were used to contextualise the quality of reporting. These elements included the clarity of the review objectives, the transparency of the search strategy, the description of the inclusion criteria, and the consistency between the aims, methods, and conclusions. No formal AMSTAR scoring was applied and individual items were not used to exclude studies.

Importantly, the methodological considerations informed the interpretation of the findings, the identification of patterns of heterogeneity, and the highlighting of evidence gaps rather than the ranking of studies or the quantitative weighting of their contribution. This approach aligns with PRISMA-ScR recommendations and reflects this review's emphasis on clinical relevance, implementation context, and patient-centred nutritional care rather than efficacy estimates.

**Supplementary Table S3. Methodological characteristics of studies included in the scoping review (n=30)**

| **Methodological category** | **No. of studies (%)** | **Specific study designs** | **Typical sample size** | **Main thematic focus** |
| --- | --- | --- | --- | --- |
| **Randomised controlled trials (RCTs)** | 5 (16,7%) | Parallel RCTs, crossover RCTs | 34–110 | Oral supplementation, intradialytic parenteral nutrition, structured dietary interventions, dietitian-led education |
| **Non-randomised interventional studies** | 3 (10.0%) | Prospective interventions, open-label trials | 10–64 | Prebiotics, antioxidant dietary strategies, creatine supplementation |
| **Observational studies** | 6 (20.0%) | Cross-sectional studies, longitudinal cohorts, retrospective and survey-based studies | 38–180 | Nutritional status, dietary intake, PEW prevalence, inflammatory burden, functional outcomes |
| **Qualitative studies** | 2 (6.7%) | Semi-structured interviews, qualitative analysis | 35–129 | Patient and caregiver experiences, perceived barriers and facilitators to dietary adherence |
| **Diagnostic accuracy study** | 1 (3.3%) | Validation studies | 148 | Muscle ultrasound for sarcopenia assessment |
| **Systematic reviews and meta-analyses** | 4 (13.3%) | Systematic reviews, meta-analyses of RCTs | 796–11,209 | Dietary adherence, probiotics/symbiotics, omega-3 supplementation |
| **Narrative and integrative reviews** | 9 (30.0%) | Narrative reviews, integrative reviews | Not applicable | Conceptual models, nutritional assessment frameworks, personalised nutrition approaches |

RCT: randomised controlled trial; HD: haemodialysis; PEW: protein–energy wasting

**Supplementary Table S4.** **Population characteristics**

| **Population feature** | **Description** |
| --- | --- |
| Age group | Adults (≥18 years); several studies focused specifically on older and frail haemodialysis patients |
| Clinical setting | Predominantly outpatient haemodialysis units |
| Nutritional status | Broad spectrum, including patients with PEW, sarcopenia, frailty and those at nutritional risk |
| Additional populations | Caregivers and healthcare professionals included in selected qualitative and observational studies |
| Dialysis vintage | Variable across studies; frequently reported as a modifier of nutritional risk |

**Supplementary Table S5. Geographic distribution of included studies**

| **Region / Continent** | **Countries included** | **Number of studies (n)** | **Percentage (%)** |
| --- | --- | --- | --- |
| Asia | Thailand, Taiwan, Japan, China, India, Lebanon, Palestine | 12 | 40.0 |
| Europe | Spain, Germany, Netherlands, Italy, Poland, Belgium¹, Greece¹, Sweden¹ | 9 | 30.0 |
| North America | United States, Mexico | 3 | 10.0 |
| South America | Brazil, Chile | 3 | 10.0 |
| Africa | Egypt, Ethiopia | 2 | 6.7 |
| Oceania | New Zealand | 1 | 3.3 |

¹One multicentre study (Mesa-Gresa *et al.,* 2023) included centres across Spain, Sweden, Belgium, and Greece.

**Supplementary Table S6. Mapping of outcomes assessed across analytical domains in included studies (n=30)**

| **Outcome domain** | **Description** | **Number of studies (n)** | **Representative outcomes / measures** |
| --- | --- | --- | --- |
| **Nutritional biomarkers** | Biochemical indicators traditionally used to assess nutritional status and metabolic control | 15 | Serum albumin, prealbumin, total protein, nPCR, BMI |
| **Inflammation and oxidative stress** | Markers reflecting systemic inflammation and oxidative burden associated with PEW and adverse outcomes | 9 | CRP, IL-6, oxidative stress markers, antioxidant capacity |
| **Dietary intake and diet quality** | Quantitative and qualitative assessment of dietary intake, food patterns and adherence | 13 | Energy/protein intake, phosphorus and potassium intake, plant-based protein proportion, ultra-processed food consumption |
| **Body composition** | Structural assessment of muscle and fat compartments beyond BMI | 11 | Lean mass (DXA, BIA), muscle thickness (ultrasound), fat-free mass |
| **Functional outcomes** | Measures of physical performance and functional capacity | 7 | Handgrip strength, physical performance tests, functional capacity scores |
| **Patient-reported outcomes** | Subjective outcomes reflecting patient experience and perceived benefit | 6 | Quality of life, vitality, appetite, self-efficacy |
| **Dietary adherence and behaviour** | Behavioural and psychosocial determinants influencing nutritional management | 13 | Adherence to diet/fluid restrictions, self-management, motivation |
| **Psychosocial and experiential factors** | Emotional, cultural and social dimensions of nutritional care | 8 | Depression, emotional burden, cultural barriers, caregiver involvement |
| **Morphofunctional assessment tools** | Integrated tools combining nutritional, inflammatory and functional dimensions | 8 | MIS, SGA, MF-BIA, muscle ultrasound |
| **Clinical outcomes** | Hard or intermediate clinical endpoints | 5 | Hospitalisation, mortality, PEW prevalence, dialysis tolerance |
| **Implementation and organisational factors** | Contextual elements affecting feasibility and sustainability of nutritional care | 7 | Availability of dietitians, interdisciplinary coordination, care models |

BMI, body mass index; BIA, bioelectrical impedance analysis; CRP, C-reactive protein; DXA, dual-energy X-ray absorptiometry; HD, haemodialysis; MF-BIA, multifrequency bioelectrical impedance analysis; MIS, Malnutrition–Inflammation Score; nPCR, normalised protein catabolic rate; PEW, protein–energy wasting; QoL, quality of life; SGA, Subjective Global Assessment.
